# Spatial pattern of genetic diversity in field populations of *Fusarium incarnatum‐equiseti* species complex

**DOI:** 10.1002/ece3.7738

**Published:** 2021-06-01

**Authors:** Sephra N. Rampersad

**Affiliations:** ^1^ Dept. of Life Sciences Faculty of Science and Technology The University of the West Indies St. Augustine Trinidad and Tobago, West Indies

**Keywords:** FIESC, genetic structure, population genetics

## Abstract

*Fusarium* is associated with a number of wilt, blight, scab, and rot diseases in a range of economically important staple food crops worldwide. An assessment of the genetic structure and population stratification of *Fusarium incarnatum‐equiseti* species complex (FIESC) pathogen populations is important to understand the evolutionary potential of such populations in adapting to environmental change. Based on intersimple sequence repeat polymerase chain reaction (ISSR‐PCR), it was found that the pathogen population was structured into three genetic clusters for which genetic differentiation was higher within than among populations. There was high intrapopulation genetic diversity for population 1 (94.63%) which consisted largely of isolates collected from North Trinidad. Populations 2 and 3 had a low level of admixture among the populations based on overall population differentiation. Population 1 accounted for the highest amount of genetic variation (95.82%) followed by populations 2 and 3. Population stratification was reflected in the dendrogram topology, which consisted of three main genetic clusters and which coincided with the outcome of Bayesian and PCoA analyses. The populations were isolated by distance, and Voronoi tessellations indicated physical or structural barriers to gene flow which contributed to restricted admixture between two of three populations. These findings suggest a high evolutionary potential for this FIESC pathogen population, the implications of which directly affect disease management strategies.

## Graphical abstract

High evolutionary potential for FIESC pathogen population directly affects disease management strategies in bell pepper.

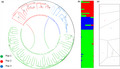

## 1 INTRODUCTION

*Fusarium* is among the most studied groups of plant‐pathogenic fungi, and member species are associated with a number of wilt, blight, scab, and rot diseases in a range of economically important staple food crops worldwide (Dean et al., 2012). This genus is large and comprises a membership of more than 1,500 species many of which are grouped into 23 defined *Fusarium* species complexes (Summerell, 2019). Certain *Fusarium* species, including *Fusarium incarnatum‐equiseti* species complex (FIESC), produce a range of mycotoxins; of notable importance are the trichothecenes, which pose a serious risk to domestic animal and human health (Desjardins, 2006). Some species are also responsible for opportunistic or secondary infections (fusariosis) in immunocompromised humans in clinical settings (Nelson et al., 1994; Nucci & Anaissie, 2007; O'Donnell et al., 2013; Short et al., 2011). Such trans‐kingdom pathogenicity with the proven ability to develop cross‐resistance to azole fungicides presents a major public health concern and contributes to the estimated billion‐dollar healthcare burden (Ananda‐Rajah et al., 2011; Benedict et al., 2019; Brown et al., 2012).

Adoption of strategies for integrated management of *Fusarium* diseases in plants remains a challenge due to the complexities of target pathosystems and the development of chemical resistance in pathogen populations (Jiménez‐Díaz & Jiménez‐Gasco, 2011). Inadvertent introductions with subsequent emergence of *Fusarium* diseases in new geographical areas further compound the adoption of integrated management methods and mitigation of the risk of mycotoxin contamination (Medina et al., 2017). New introductions may arise inadvertently through anthropogenic movement via seed and grain trade (Lee et al., 2015). The impact of long‐range, aerial dispersal of pathogens on global distribution of plant diseases remains a critical epidemic‐associated factor (Brown & Hovmoller, 2002; Schmale & Bergstrom, 2003; Schmale et al., 2006). Palmero et al. (2011) and others reported that spores (ascospore and macroconidia) of *Fusarium*, with differing levels of pathogenicity, are able to cross the Atlantic carried by winds from the Sahara (Africa) to the Caribbean, Europe, and the Mediterranean (Griffin et al., 2003; Prospero et al., 2005; Prospero & Lamb, 2003). As much as 2 million metric tons of dust are deposited each year (Guerzoni et al., 1997). Selection pressures work cooperatively to favor the predominance of certain introduced genotypes that are well‐adapted to available host(s) and their new environment (Summerell et al., 2007).

*Fusarium* populations are dynamic in their responses to environmental cues, the consequence of which include increased yield losses, reduction in quality, or changes in the mycotoxins produced (Valverde‐Bogantes et al., 2019). An assessment of the genetic structure and population stratification of pathogen populations is important to understanding the evolutionary potential of such populations in adapting to environmental change, the effects of selection pressures, and the relative impact of the various drivers of evolutionary change, for example, genetic drift, mutation, gene flow, isolation with the ultimate selection of those genotypes with increased biological fitness, and subsequent adaptive advantage (McDonald & Linde, 2002). Genetic structure would also reveal signatures of sexual recombination and clonal spread of isolates within and among populations (Heule et al., 2017). A population with highly pathogenic isolates and different trichothecene chemotypes can replace or dominate another (Fulcher et al., 2019; Kelly et al., 2015).

An efficient approach to estimating the genetic structure of pathogen populations is to randomly screen representatives using selectively neutral molecular marker loci on geographically defined samples collected under a hierarchical sampling strategy (Grünwald et al., 2017). ISSR‐PCR (Inter Simple Sequence Repeat PCR/single primer amplification reactions (SPAR)/microsatellite‐primed PCR (MP‐PCR)) relies on amplification of a target region of nucleotide sequences defined by anchored or nonanchored SSR (simple sequence repeats) homologous primers (Zietkiewicz et al., 1994). ISSR‐generated polymorphisms are analyzed as dominant genetic markers and are widely used over other polymorphism generators, for example, RAPD—randomly amplified polymorphic DNA and AFLP—amplified fragment length polymorphism, to determine genetic variations due to cost effectiveness, reproducibility, generation of a high level of polymorphism because they are inherently very variable, and a priori knowledge about the target sequences is not required (Ng & Tan, 2015). Further, because ISSRs are ubiquitously distributed across the genome, the entire genome under study is theoretically represented in the resulting multilocus data rather than a few, user‐selected gene regions. A number of studies have characterized different *Fusarium* populations in plants using ISSR genetic markers (Akbar et al., 2018; Altinok et al., 2018; Dinolfo et al., 2010; Hamdi et al., 2019; Mishra et al., 2003, 2004; Nawade et al., 2017; Singh et al., 2019; Thangavelu et al., 2012). However, Lee et al. (2015) referred to a paucity of similar analyses of European FHB populations using neutral markers which result in limited interpretations of population dynamics of FHB pathogens on this continent.

Trinidad (10.6918°N, 61.2225°W) is situated off the northeast coast of South America at the southernmost end of the Lesser Antilles in the Caribbean and has a land size of 4,768 km^2^ of which <10.56% is arable land. Bell pepper is among the top 10 agricultural commodities in the country. Cultivation occurs year‐round to meet local and regional export demands. The average area cultivated by an individual grower is approximately 0.31 hectares (CARDI, Caribbean Agricultural Research, & Development Institute, 2013). In 2015, it was reported that 245 farmers cultivated sweet pepper in Trinidad with the highest concentration occurring in the North (49%) (PROPEL, Promotion of Regional Opportunities for Produce through Enterprises, and Linkages (PROPEL), 2015). *Fusarium incarnatum‐equiseti* (FIESC) species complex is among the major fungal pathogens that cause fruit rot disease which leads to high production cost, severe economic losses, and, ultimately, inconsistent production (Ramdial et al., 2016, 2017). The FIESC includes over 30 recognized phylogenetic species (phylo‐species) which have been characterized through the steadfast efforts of a number of research groups, worldwide (Aoki et al., 2014; Maryani et al., 2019; O'Donnell et al., 2009, 2010; Santos et al., 2019; Villani et al., 2016; Wang et al., 2019). Correct identification of *Fusarium* haplotypes is carried out according to published guidelines (Geiser et al., 2013; O'Donnell et al., 2015; O'Donnell et al., 2010) in addition to the recommendations provided by the CBS‐KNAW Fungal Biodiversity Centre's *Fusarium* MLST database (http://Fusarium.mycobank.org/). Recently, Xia et al. (2019) published a revision of this species complex where the haplotypes identified in Trinidad can now be assigned species names in addition to the numerical haplotype classification: *Incarnatum* species are *Fusarium irregulare* (FIESC 15), *Fusarium sulawesiensis* (FIESC 16), and *Fusarium hainanense* (FIESC 26); *Equiseti* species are *Fusarium ipomoeae* (FIESC 1) and *Fusarium longifundum* (FIESC 11). *Fusarium sulawesiensis* (FIESC 16) is the most common species, and *F. ipomoeae* (FIESC 1) is the least common species isolated from infected Trinidad bell pepper fruit (Villafana & Rampersad, 2020).

In view of the severity of the disease in bell pepper in Trinidad, the dispersal of fusaria via Saharan dust storms, poor management of the disease, and inability to reduce pathogen populations through chemical means, there are several biological questions that must be answered through population genetics analyses for example: Are FIESC populations differentiated? Is there gene flow among populations? Are populations clonal, sexual, or mixed? It is hypothesized that FIESC populations in bell pepper fields would be connected by some level of contemporary gene flow partly because seed and seedlings are commonly sourced and the comparatively short distance (km) among fields may not facilitate isolation by distance. As such, the level of population differentiation and genetic diversity may vary according to region, that is, North and South Trinidad but not necessarily at a finer scale, that is, according to field location. One expectation is that the genetic structure of pathogen populations would be subjected to local extinction‐recolonization events as the host is harvested, destroyed, and then re‐planted during the growing season. Trans‐Atlantic spore movement which although stochastic may be representative of unrestricted gene flow and leads to less differentiated populations. Related to these expectations is the “monoculture effect” where the association between low host species diversity and high disease incidence is a result of high homogeneity in the host population (King & Lively, 2012; Thrall et al., 2003). Based on these hypotheses, the objectives of this study were to (a) estimate the genetic diversity and population structure of FIESC in Trinidad, (b) characterize genetic differentiation within and among populations, (c) determine whether populations were isolated by distance.

## 2 RESULTS

### 2.1 ISSR markers

**FIGURE 1:**
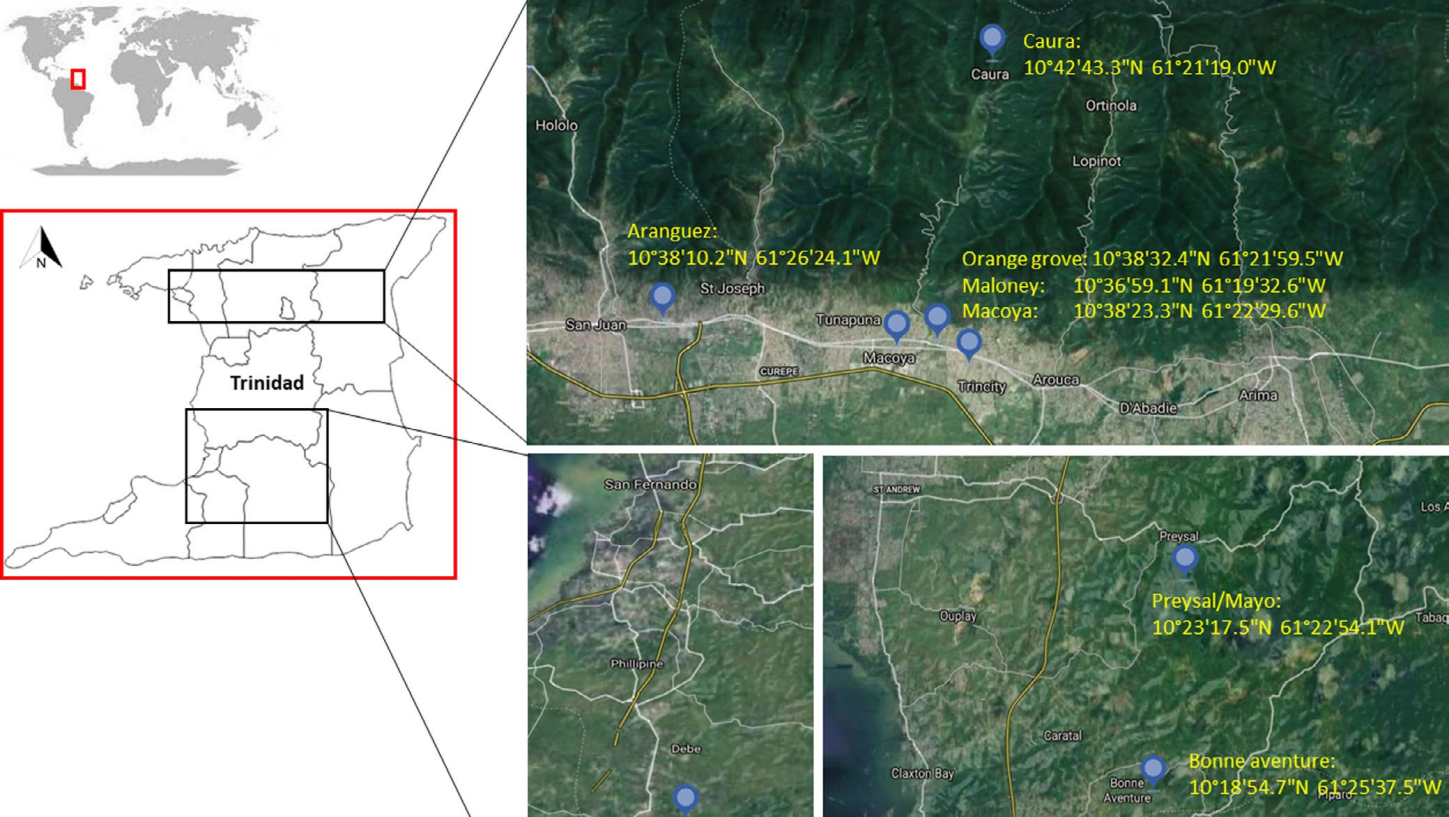
Sampling locations in Trinidad are given as the name of the region followed by their respective latitude/longitude coordinates. Trinidad is the southernmost island in the Lesser Antilles and is situated ~11km off the northeastern coast of Venezuela in South America

Sampling sites are indicated in Figure 1, and isolates collected and used in the analysis are outlined in Table S1. Only those individuals that produced a polymorphic banding pattern for the ISSR primers and for which the interpretation of the banding pattern was unequivocal were retained in the final data set. Fragments with the same molecular weight were considered to be the same locus. In total, 335 polymorphic loci were generated from ISSR‐PCR using the five ISSR primers selected from a screen of 22 ISSR primers (Table S2).

### 2.2 Population stratification

**FIGURE 2:**
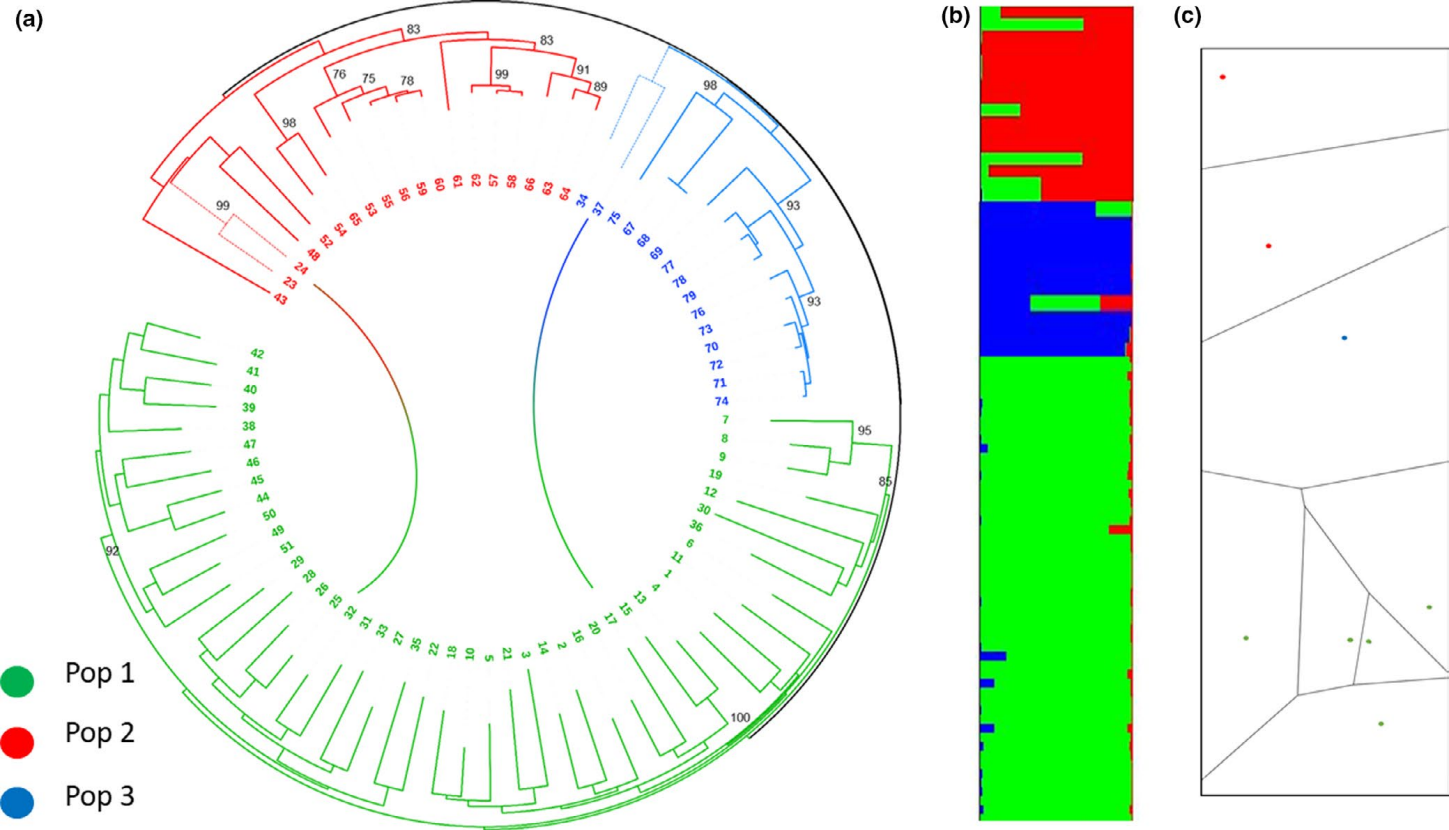
(a) Dendrogram inferred from Nei's genetic distance matrix using the heuristic approach of the neighbor‐joining algorithm with 1,000 bootstrapped replicates. Numbers are the nodes indicate bootstrap values ≥75%. Colors correspond to STRUCTURE‐inferred optimal *K* clusters. (b) Bar plot of individual Q matrix coefficients for FIESC isolates from assignment tests carried out in STRUCTURE with *K* = 3 as the optimal number of populations. Each vertical bar represents an individual isolates, and bars are divided based on the probability of assignment of each individual to a given population. (c) Hard clustering membership based on genetic data and geographical distance. Colors correspond to STRUCTURE‐inferred optimal *K* clusters

Assignment of isolates was carried out by Bayesian clustering implemented in STRUCTURE software. Posterior probabilities calculated by STRUCTURE and STRUCTURE HARVESTER indicate a population structure at *K* = 3 genetic clusters (Figure 2). The overall estimated proportion of membership of the samples to each of the three inferred genetic groups was population 1 accounted for the highest amount of variation (95.82%) in the dataset; 25.37% and 12.54% of the total variation were attributed to populations 2 and 3, respectively. Assignment of isolates to putative genetic clusters is illustrated in Figure 2: Population 1 (green) consisted exclusively of isolates collected from North Trinidad; the membership of population 2 primarily consisted of isolates collected from South Trinidad; and population 3 comprised of isolates collected from fields located in the Mayo, Caura, and Aranguez regions.

**FIGURE 3:**
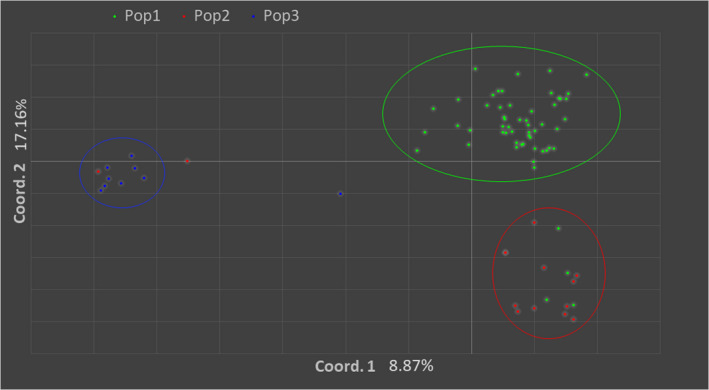
A principal coordinate (PCoA) plot of the first two coordinates calculated using Nei's D genetic distance among FIESC isolates in Trinidad. Colors correspond to STRUCTURE‐inferred optimal K clusters

PCoA was carried out to visualize genetic similarity among individuals to substantiate inferences of interindividual and intergroup relationships. PCoA results were concordant with Bayesian cluster analysis implemented by STRUCTURE and POPS in the apparent distribution of isolates into three main populations. The first two principal coordinates in the PCoA of Nei's *D* distance explained 8.87% and 17.16% of the total genetic variation, respectively. The PCoA showed that the isolates from North formed a distinct genetic cluster but there were isolates from the North which were more similar to those in the South genetic cluster than the North. Also, there were isolates from the South that were more similar to isolates from Mayo (Figure 3).

The total genetic variation was partitioned into within‐ and among‐population components. AMOVAs (Table 1) revealed that the estimated genetic variation in the dataset was largely due to within‐population variation (81%) as opposed to among‐population variation (19%) at *p* < 0.001.

**TABLE 1:** Analysis of variance within‐ and among deduced populations

Source	*df*	SS	MS	Est. Var.	%
Among Pops	2	181.665	90.832	4.089	19%
Within Pops	76	1,340.538	17.639	17.639	81%
Total	78	1,522.203		21.728	100%

Abbreviations: %, percentage variation; *df*, degrees of freedom; Est. Var., estimated variation; MS, mean sum of square; SS, sum of squares.

### 2.3 Genetic clusters

Data from the genetic distance matrix were analyzed using the NJ algorithm (Figure 2). Population stratification was reflected in the dendrogram topology, which consisted of three main genetic clusters and which coincided with the outcome of Bayesian and PCoA analysis. In genetic cluster 2 (red), there were two isolates (23 and 24—denoted by dashed branch lines) that were collected from North but placement occurred with South isolates. Similarly, two isolates (34 and 37—denoted by dashed branch lines) were collected from the North but were more genetically similar to isolates collected from Mayo (blue). These connections were depicted by the gradient lines extending from the corresponding nodes in population 2 (red) and population 3 (blue) to population 1 (green).

### 2.4 Gene diversity

The overall mean gene diversity (*H_T_*) was 0.1006, and the mean within‐population gene diversity (*H_S_*) was 0.0729. Population 1 had the highest gene diversity (*h* = 0.1202) followed by population 2 (*h* = 0.0599) and population 3 (*h* = 0.0386). Demographic variables such as population size appeared to be a factor in the gene diversity indices for populations. Pairwise comparisons of Nei's unbiased measures of genetic identity and genetic distance estimated for three inferred populations are given in Table 2. The overall fixation index G_ST_, as it relates to nearness to fixation, for the Trinidad population (*G*_ST_) was 0.2755 which indicated that the total genetic variation mainly exists within the population.

**TABLE 2:** Nei's genetic identity (above diagonal) and genetic distance (below diagonal)

Pop	1	2	3
1	***	0.9702	0.9565
2	0.0303	***	0.9410
3	0.0445	0.0609	***

### 2.5 Isolation by distance

The correlation between genetic distance and logarithmic geographic distance was significant (*p* < 0.01) after 999 permutations based on Mantel's test (Figure S1). The spatial clustering of Voronoi tessellation model behaved similar to the Pritchard et al. (2000) model from which the Q matrix and structure plot was based. Although both models utilize Bayesian statistical inference, the assumptions of each approach are different. POPS data indicated genetic differentiation by distance with physical and or structural barriers to gene flow among the three populations (Figure 2c).

### 2.6 Indirect estimate of gene flow

There was a low level of gene flow among the three populations where *Nm* = 1.3147 which indicated limited gene exchange among populations in this study. *Nm* > 4 indicates extensive gene flow among populations. If *Nm* > 1, there is enough gene flow to negate the effects of genetic drift, and if *Nm* > 4, then local populations belong to one panmictic (randomly mating) population (Wright, 1931).

### 2.7 Index of association

In the index of association tests (Weir, 1979), the rBarD value is expected to be zero if populations are freely recombining (sexual reproductive mode) and greater than zero if there is association between alleles (i.e., clonality or asexual reproductive mode). The rBarD statistic is considered to be a more robust measure of association (Agapow & Burt, 2001). There was support for the hypothesis that alleles are linked across loci (*p* < 0.05) in the three deduced populations. These results suggest significant clonality within each of the three populations with population 3 having the highest *I_A_* value and population 1 having the lowest *I_A_* value (Table 3).

**TABLE 3:** Index of association statistics

Pop	*N*	*I_A_*	rBarD	rBarS
all	78	1.09096	0.003693	−0.01174
1	53	0.53704	0.001866	−0.01292
2	14	6.29444	0.089178	0.06540
3	11	12.2866	0.207408	−0.17377

## 3 DISCUSSION

The aim of this study was to understand and compare the extent and distribution of genetic variation within pathogenic *Fusarium* populations, the level of population subdivision, and the interconnectivity of these and other evolutionary factors involved in population dynamics. This information will ultimately assist in predicting the pathogenic potential of different *Fusarium* species with clear implications for the development of integrated, efficient, and sustainable strategies for disease management (McDonald & Linde, 2002; Peever et al., 2000). To achieve this primary aim, this study was structured around interconnected hierarchical levels to explain the population genetics of FIESC infecting bell pepper in Trinidad. Three genetic clusters, supported by AMOVA, PCoA, and Bayesian model‐based clustering, largely coincided with the geographic location of fields with the exception of a few isolates. The number of genetic clusters detected by Bayesian clustering algorithms can be biased by the choice of a particular sampling strategy (Schwartz & McKelvey, 2009; Waples & Gaggiotti, 2006), the sample size, and the number of usable polymorphisms (Fogelqvist et al., 2010; François & Durand, 2010). Although different model selection criteria were applied in order to determine values of *K* that optimally describe the data for each algorithm (Dinolfo et al., 2017) (Bayesian model implemented in STRUCTURE and deviance information criterion (DIC) implemented in POPS), it is possible that a finer degree of substructuring as a result of higher genetic variation in genetic clusters 2 and 3 may be detected with larger sample sizes.

In this study, there was high intrapopulation genetic variation for population 1 (94.63%) which consisted largely of isolates collected from North Trinidad compared to populations 2 and 3, perhaps due to larger sample size. It was reported that for *Fusarium poae* isolates, genetic variability was explained by differences within rather than between Argentinean and English populations (Dinolfo et al., 2017). Similarly, in several different studies of *Fusarium graminearum* populations in Canada, genetic diversity was distributed within populations and not among populations (Miedaner et al., 2001, 2003; Mishra et al., 2004). Akbar et al. (2018) purported that monoculture cropping may explain cases of low genetic diversity of *Fusarium equiesti* populations. However, Dinolfo et al. (2017) also reported that geographic isolation, ecological conditions, and crop rotation systems may not have a significant effect on the genetic variability and distribution of *F. poae* isolates which may suggest pathosystem‐specific interactions.

There was a significant correlation between genetic distance and geographical distance based on two models assessed in this study. Infected seeds and/or host plants would have been distributed to different growing areas through anthropological agricultural activities. Mishra et al.(2004) suggested that highly diverse populations may arise from free movement of the pathogen as wind‐borne ascospores and/or via anthropogenic exchange of infected plant material. Re‐colonization by pathotypes during successive growing seasons is related to the geographical proximity to the source population in which a given pathotype was present (Thrall et al., 2003).

Rosenberg et al. (2005) noted that there are a number of variables that influence the clustering of individuals using genome‐wide markers, for example, sample size, number of loci, number of clusters, assumptions about correlations in allele frequencies across populations, and the geographic distribution of samples. The relationship between genetic and geographic distance should not be an artifact of the sampling scheme, but should represent discontinuity of pairwise genetic distances of two populations on opposite sides of a structural or physical barrier, when compared with pairwise genetic distances of two populations on the same side of the same barrier (Rosenberg et al., 2005). Usually, a minority of individuals that exist in intermediate/neighboring geographic locations can have mixed membership in the main genetic clusters (Rosenberg et al., 2005).

Naef and Défago (2006) studied pathogenic and saprophytic isolates of *F. graminearum* in Germany and asserted that sexually produced, wind‐dispersed ascospores supported gene flow which (a) prevented substructuring within the saprophytic *F. graminearum* population, (b) enabled mixing between saprophytic and pathogenic populations of *F. graminearum* located in fields 100 km apart, and (c) resulted in shared genetic similarity of saprophytic and pathogenic *F. graminearum* population. Schmale and Bergstrom (2003) concluded that the diverse atmospheric populations of *Giberella zeae* (Schwein.) Petch (anamorph *F. graminearum* Schwabe) could have originated from several locations over large geographical area and were transported through the atmosphere over long distances. Attendant to this finding, however, Cowger et al. (2020) reported that *F. graminearum* isolates around the world nevertheless show significant geographic substructure and that even populations that are in geographic proximity can show distinct substructuring.

Gene flow breaks down the boundaries that could otherwise isolate populations and may be especially important for plant pathogens in agroecosystems because it is the process that introduces new genetic variation into agricultural fields distant from the site of the original mutation (McDermott & McDonald, 1993). Estimated gene flow in the FIESC populations in Trinidad was <4 but greater than 1 (*Nm* = 1.3147). According to Wright (1952), if gene flow is greater than 4, the individuals are considered to be part of a single population. Gene flow was identified as an important contributor to maintaining the high genetic diversity in populations of *F. graminearum* through the dispersal of sexual and asexual propagules (Gale et al., 2002; Mishra et al., 2004; Zeller et al., 2004). It is worth noting that various factors affect gene flow, for example, host availability and climatic events (environmental), reproductive, migratory, and dispersal mechanisms (biological), and modes of reproduction (genetic) (Rogers et al., 1999).

Linkage disequilibrium (LD) throughout the genome is indicative of population history, the breeding system, and the pattern of geographic subdivision in populations (Slatkin, 2008). Pathogens whose survival depends on "mixed" mating/reproduction systems, that is, both sexual and asexual reproduction, tend to have high genetic diversity. The FIESC isolates in Trinidad appeared to be freely recombining as evidenced by the rbarD statistic which was less than zero for each of the three genetic clusters identified and for which population 1 had the highest genetic variation and the highest rbarD value compared to the other two populations. Mishra et al. (2003) reported that index of association data suggested a mixed mode of reproduction for *Fusarium culmorum*. This finding contradicts the assertion that a sexual stage of *F. culmorum* has never been observed (Leslie & Summerell, 2006). Sexual recombination was found to be frequent in *F. graminearum* populations from western Canada which contributed to high within‐population genetic diversity (Mishra et al., 2004). Genetic drift, selective forces acting within populations, and population admixture can also cause LD between genetic markers (Remington et al., 2001).

The implications of high evolutionary potential within a given pathogen population directly affect disease management (McDonald & Linde, 2002). High genetic diversity of populations of *F. graminearum, Fusarium pseudograminearum*, and *F. culmorum* enabled greater adaptive flexibility of these pathogens exemplified by population shift from *F. culmorum* to *F. graminearum* in the Netherlands (Waalwijk et al., 2003), the United Kingdom (Jennings et al., 2004), northern Germany (Miedaner et al., 2008), and in the western provinces of Canada (Clear & Patrick, 2019; Mishra et al., 2004, 2006). Changes in pathogenicity have been reported in relation to a shift from DON producers to a higher proportion of NIV‐producing *F. graminearum* and *F. culmorum* in Europe (Waalwijk et al., 2003). *Fusarium solani* was reported to be an important pathogen of bell pepper fruit in Trinidad (Ramdial & Rampersad, 2010) but has since been largely replaced by the now predominant FIESC with demonstrated higher genetic variation and pathogenicity.

## 4 MATERIALS AND METHODS

### 4.1 Isolate collection

Bell pepper fruits that were symptomatic of *Fusarium* fruit rot (Ramdial et al., 2017) were collected from the main pepper production areas in Trinidad. This involved a country‐wide survey that was carried out from 2014 to 2017 (Table 1; Figure 1). In total, 79 FIESC isolates were collected and their identity was confirmed by multilocus sequence comparisons in a separate study (Villafana & Rampersad, 2020). There were no a priori assumptions about the number of populations or represented by these Trinidad isolates. One isolate (79) was identical to isolate 78 and thus was not included in tests of genetic diversity, AMOVA, and association for which the dataset consisted of 78 isolates.

### 4.2 ISSR profiling

Total genomic DNA was extracted from single‐spore cultures of FIESC isolates grown in potato dextrose broth for 7 days in the dark using the Maxwell‐16^®^ automated DNA extraction kit in accordance with the manufacturer's instructions (Promega Corporation). Ten FIESC isolates were used in a preliminary screen to determine those ISSR primers that enabled generation of a high number of polymorphic (>100) and reproducible markers (identically sized bands in two experiments) that could be used to generate polymorphic DNA fragments for all isolates. Twenty‐two ISSR primers (Integrated DNA Technologies Inc.) were screened which included an assessment of optimal primer annealing temperature by gradient annealing temperature analysis (Tables S1 and S2). These primers were also used in another study to determine the genetic variability of *Colletotrichum* species in Trinidad (Rampersad, 2013). The optimized PCR mixture (25 μl total volume) contained 12.5 μl of GoTaq^®^ Green Master Mix (Promega), 0.5 μl (10 μM) of each primer (Integrated DNA Technologies Inc.), 6.5 μl of nuclease‐free water (Promega), and 5 μl of DNA template. Standard PCR amplification conditions were an initial denaturation of 5 min at 95℃; followed by 35 cycles of 30 s at 95℃, 30 s at 41℃ to 60℃, and 90 s at 72℃, with a final extension of 5 min at 72℃. The PCR reactions were carried out on Thermal Cycler 2720 (Thermo Scientific). PCR products were separated on 1.4% agarose gels stained with ethidium bromide. PCR reactions were conducted twice to confirm reproducibility of the fingerprints. Those primers that generated reproducible, distinct, polymorphic bands in repeated experiments were selected for subsequent amplification of genomic DNA from all isolates. Bands were computationally scored by gel image analysis using GelAnalyzer 2010a software (http://www.gelanalyzer.com). Only consistently scored bands of size range 100–2,500 bp were used under the assumption that each band represented a distinct locus and amplicons sharing the same molecular weight shared the same locus. Scored bands were transformed into a binary character matrix (1 = presence; 0 = absence).

### 4.3 Data analysis

The assignment of each isolate to a particular population or genetic cluster was carried out STRUCTURE v. 2.3.4 (Pritchard et al., 2002) which applies a Bayesian clustering approach using Markov Chain Monte Carlo (MCMC) estimation. No a priori assumptions about the number of populations or subgroups were made and the analyses were run for 50,000 MCMC replications after an initial burn‐in period of 10,000 generations. Membership probabilities of individuals to a population were assessed for *K* = 1 to *K* = 5 where *Δ*K is predicted as that number of populations or genetic groups with the highest likelihood in the dataset. STUCTURE HARVESTER (http://taylor0.biology.ucla.edu/structureHarvester; Earl & Holdt, 2012) confirmed the number of populations with the highest likelihood according to method of Evanno et al. (2005). The POPS (Prediction of Population Genetic Structure (Jay et al., 2015)) software uses a “TESS‐like interface” to compute, assign, and predict individual membership probabilities and geographical clustering of individuals based on user‐defined multilocus genetic and geographical data. No predefined population was assumed. Bayesian inference enables identification of genetic and geographical discontinuities in mixed populations (François et al., 2006; Guillot et al., 2005). Different localities are plotted as spatial domains, and populations occupy a particular subdomain under the assumption that each subdomain is connected in space using convex polygons; as such, a Voronoi mosaic is produced. To determine the optimal number of populations, *K*, the deviance information criterion (DIC) was compared to determine the best fit of a set of Bayesian hierarchical models (Spiegelhalter et al., 2002).

GenAlEx version 6.5 (Genetic Analysis in Excel (Peakall & Smouse, 2006)) was used to produce a genetic distance matrix for which analysis of molecular variance (AMOVA) was carried out. Principal coordinates analysis (PCoA) was also conducted to visualize separation of the isolates into discrete genetic clusters. An unrooted, neighbor‐joining (NJ) dendrogram was constructed to represent the genetic distances among the population using MEGAX (Molecular Evolutionary Genetic Analysis software (Kumar et al., 2018)). Bootstrapping was carried out in PAST 4 (Paleontological statistics (Hammer et al., 2001)). Nei's gene diversity (h) (Nei, 1973), allele frequencies, and pairwise comparisons of genetic diversity for each inferred population were determined using POPGENE version 1.32 (Yeh et al., 1999) with 1,000 replicates. Isolation by distance among populations was evaluated using Mantel tests between log‐transformed geographic distance and genetic distance in GenAlEx 6.5. Significance of the correlation was tested assessing the *p*‐value from 999 permutations. Indices of multilocus linkage disequilibrium were determined in MULTILOCUS (https://agapow.net/software/multilocus/; Agapow & Burt, 2001).

## CONFLICT OF INTEREST

The author declares no conflict of interest.

## AUTHOR CONTRIBUTION

**Sephra N. Rampersad:** Conceptualization (lead); Data curation (lead); Formal analysis (lead); Funding acquisition (lead); Investigation (lead); Methodology (lead); Project administration (lead); Resources (lead); Software (lead); Supervision (lead); Validation (lead); Visualization (lead); Writing‐original draft (lead); Writing‐review & editing (lead).

## Supporting information

Supplementary Material

## Data Availability

Upon acceptance of this manuscript, all genotyping data will be archived and made available in the Dryad data repository (https://doi.org/10.5061/dryad.w6m905qpm).
