# Supplementary material for: Spatial pattern of genetic diversity in field populations of Fusarium incarnatum‐equiseti species complex
Source: Ecol Evol. 2021 Jun 1;11(13):9010–20. doi: 10.1002/ece3.7738 (PMC8258202; doi:10.1002/ece3.7738)
Supplement: Supplementary file 1 — Supplementary Material [file ECE3-11-9010-s001.docx]

**Spatio-genetic pattern of diversity in field populations of *Fusarium incarnatum-equiseti* species complex**

**Sephra N. Rampersad***

The University of the West Indies, Faculty of Science and Technology, Dept. of Life Sciences, St. Augustine. Trinidad and Tobago – West Indies.

*Corresponding author: Sephra Rampersad; sephra.rampersad@sta.uwi.edu

**Supplemental files**

**Supplemental Figure S1. Plot of genetic distance (GD) vs. geographic distance according to Mantel’s test of isolation by distance**

| **Isolate #** | **MLST^1^ type** | **Species^2^** | **Field location^3^** |
| --- | --- | --- | --- |
| 1 | FIESC 26 | *F. hainanense* | OG1 |
| 2 | FIESC 16 | *F. sulawesiensis* | OG2 |
| 3 | FIESC 15 | *F. irregulare* | OG9 |
| 4 | FIESC 26 | *F. hainanense* | OG3 |
| 5 | FIESC 16 | *F. sulawesiensis* | OG4 |
| 6 | FIESC 16 | *F. sulawesiensis* | OG20 |
| 7 | FIESC 1 | *F. ipomoeae* | OG5 |
| 8 | FIESC 11 | *F. longifundum* | OG6 |
| 9 | FIESC 11 | *F. longifundum* | OG7 |
| 10 | FIESC 16 | *F. sulawesiensis* | Mal1 |
| 11 | FIESC 26 | *F. hainanense* | Mac1 |
| 12 | FIESC 11 | *F. longifundum* | OG8 |
| 13 | FIESC 15 | *F. irregulare* | OG10 |
| 14 | FIESC 16 | *F. sulawesiensis* | Mal2 |
| 15 | FIESC 15 | *F. irregulare* | OG11 |
| 16 | FIESC 15 | *F. irregulare* | OG12 |
| 17 | FIESC 26 | *F. hainanense* | Mac2 |
| 18 | FIESC 16 | *F. sulawesiensis* | Mal3 |
| 19 | FIESC 15 | *F. irregulare* | OG13 |
| 20 | FIESC 15 | *F. irregulare* | OG14 |
| 21 | FIESC 16 | *F. sulawesiensis* | Mal4 |
| 22 | FIESC 15 | *F. irregulare* | OG15 |
| 23 | FIESC 26 | *F. hainanense* | Mac3 |
| 24 | FIESC 26 | *F. hainanense* | Mac4 |
| 25 | FIESC 16 | *F. sulawesiensis* | Mal5 |
| 26 | FIESC 16 | *F. sulawesiensis* | Mal6 |
| 27 | FIESC 16 | *F. sulawesiensis* | Mal7 |
| 28 | FIESC 15 | *F. irregulare* | OG16 |
| 29 | FIESC 16 | *F. sulawesiensis* | Mal8 |
| 30 | FIESC 16 | *F. sulawesiensis* | Mal8 |
| 31 | FIESC 15 | *F. irregulare* | OG17 |
| 32 | FIESC 16 | *F. sulawesiensis* | Mal9 |
| 33 | FIESC 15 | *F. irregulare* | OG18 |
| 34 | FIESC 26 | *F. hainanense* | Mac5 |
| 35 | FIESC 16 | *F. sulawesiensis* | Mal10 |
| 36 | FIESC 15 | *F. irregulare* | OG19 |
| 37 | FIESC 26 | *F. hainanense* | Mac6 |
| 38 | FIESC 16 | *F. sulawesiensis* | Mal11 |
| 39 | FIESC 16 | *F. sulawesiensis* | Mal12 |
| 40 | FIESC 16 | *F. sulawesiensis* | Mal13 |
| 41 | FIESC 16 | *F. sulawesiensis* | Mal14 |
| 42 | FIESC 16 | *F. sulawesiensis* | Mal15 |
| 43 | FIESC 26 | *F. hainanense* | Mac7 |
| 44 | FIESC 16 | *F. sulawesiensis* | Mal16 |
| 45 | FIESC 16 | *F. sulawesiensis* | Mal17 |
| 46 | FIESC 16 | *F. sulawesiensis* | Mal18 |
| 47 | FIESC 16 | *F. sulawesiensis* | Mal19 |
| 48 | FIESC 26 | *F. hainanense* | Mac8 |
| 49 | FIESC 16 | *F. sulawesiensis* | Mal20 |
| 50 | FIESC 16 | *F. sulawesiensis* | Mal21 |
| 51 | FIESC 16 | *F. sulawesiensis* | Mal22 |
| 52 | FIESC 26 | *F. hainanense* | BA1 |
| 53 | FIESC 16 | *F. sulawesiensis* | BA2 |
| 54 | FIESC 26 | *F. hainanense* | BA3 |
| 55 | FIESC 16 | *F. sulawesiensis* | BA4 |
| 56 | FIESC 16 | *F. sulawesiensis* | BA6 |
| 57 | FIESC 16 | *F. sulawesiensis* | BA7 |
| 58 | FIESC 16 | *F. sulawesiensis* | BA8 |
| 59 | FIESC 16 | *F. sulawesiensis* | BA9 |
| 60 | FIESC 16 | *F. sulawesiensis* | BA10 |
| 61 | FIESC 15 | *F. irregulare* | PE1 |
| 62 | FIESC 16 | *F. sulawesiensis* | PE2 |
| 63 | FIESC 16 | *F. sulawesiensis* | PE3 |
| 64 | FIESC 26 | *F. hainanense* | PE4 |
| 65 | FIESC 16 | *F. sulawesiensis* | PE5 |
| 66 | FIESC 16 | *F. sulawesiensis* | POSG2 |
| 67 | FIESC 26 | *F. hainanense* | POSR1 |
| 68 | FIESC 26 | *F. hainanense* | POSR2 |
| 69 | FIESC 16 | *F. sulawesiensis* | C1 |
| 70 | FIESC 16 | *F. sulawesiensis* | C2 |
| 71 | FIESC 16 | *F. sulawesiensis* | A1 |
| 72 | FIESC 16 | *F. sulawesiensis* | A2 |
| 73 | FIESC 16 | *F. sulawesiensis* | Ma1 |
| 74 | FIESC 16 | *F. sulawesiensis* | Ma2 |
| 75 | FIESC 26 | *F. hainanense* | Ma7 |
| 76 | FIESC 16 | *F. sulawesiensis* | Ma3 |
| 77 | FIESC 16 | *F. sulawesiensis* | Ma4 |
| 78 | FIESC 16 | *F. sulawesiensis* | Ma5 |

**Supplementary Table S1. Isolate data**

^1^ MLST type: Multilocus sequence type assignation. One isolate, 79, was identical to another isolate, 78, and as such, 79 was removed from further genetic analysis.

^2^ Field location: A – Aranguez; BA - Bonne aventure; C – Caura; Ma – Mayo; Mac – Macoya; Mal – Maloney; OG - Orange grove; PE – Penal; POS - Port of Spain. The number after each location code refers to the field sampled.

^3^Species designations according to Xia et al.^53^

| **ISSR primer name^a^** | **ISSR primer sequence** | **Ta (°C)^b^** | **Anchor/**  **Anchorless primer** |
| --- | --- | --- | --- |
| *P1 | (AG)8 | 43.4 | Anchorless |
| P2 | (AC)8 | 47.4 | Anchorless |
| P3 | (CAG)5 | 47.4 | Anchorless |
| P4 | (CAA)5 | 33.7 | Anchorless |
| *P5 | (GTG)5 | 47.4 | Anchorless |
| P6 | (CAC)5 | 47.4 | Anchorless |
| P7 | (ACA)5 | 33.7 | Anchorless |
| P8 | (AAC)5 | 33.7 | Anchorless |
| *P9 | (CCA)5 | 54.9 | Anchorless |
| P10 | (AAG)6 | 41.2 | Anchorless |
| P11 | (GTC)6 | 54.9 | Anchorless |
| P12 | (GACA)4 | 43.4 | Anchorless |
| P13 | (GGAT)4 | 44.7 | Anchorless |
| P14 | (GACAC)3 | 44.7 | Anchorless |
| P15 | (AG)8TG | 48.0 | 3′ anchor |
| P16 | (AG)8CG | 48.0 | 3′ anchor |
| P17 | (CT)8G | 48.0 | 3′ anchor |
| P18 | (CA)8GT | 48.0 | 3′ anchor |
| P19 | CTC(GT)8 | 48.0 | 5′ anchor |
| *P20 | CT(GA)8 | 48.0 | 5′ anchor |
| *P21 | BDB(ACA)5 | 48.0 | 5′ anchor |
| P22 | GAG(CAA)5 | 48.0 | 5′ anchor |

**Supplementary Table S2. Characteristics of inter-simple-sequence repeat (ISSR) primers**

^a^ Asterisk (*) indicates the five ISSR primers that were selected and ultimately used in repeated experiments.

^b^ Annealing temperature.

| **Polymorphism parameter** | **Pop 1** | **Pop 2** | **Pop 3** |
| --- | --- | --- | --- |
| No. Bands | 321 | 85 | 48 |
| No. Bands Freq. >= 5% | 168 | 85 | 48 |
| No. Private Bands | 235 | 5 | 1 |
| No. LComm Bands (<=25%) | 0 | 0 | 0 |
| No. LComm Bands (<=50%) | 0 | 0 | 0 |
| Mean h | 0.120 | 0.068 | 0.037 |
| SE of Mean h | 0.006 | 0.007 | 0.006 |
| Mean uh | 0.122 | 0.074 | 0.041 |
| SE of Mean uh | 0.006 | 0.008 | 0.007 |

**Supplementary Table S3. Band polymorphism data according to deduced population**

No. Bands = No. of Different Bands

No. Bands Freq. >= 5% = No. of Different Bands with a Frequency >= 5%

No. Private Bands = No. of Bands Unique to a Single Population

No. LComm Bands (<=25%) = No. of Locally Common Bands (Freq. >= 5%) Found in 25% or Fewer Populations

No. LComm Bands (<=50%) = No. of Locally Common Bands (Freq. >= 5%) Found in 50% or Fewer Populations

h = Diversity = 1 - (p^2 + q^2)

uh = Unbiased Diversity = (N / (N-1)) * h

Where for Haploid Binary data, p = Band Freq. and q = 1 - p.
